# [Corrigendum] MicroRNA-222-3p promotes tumor cell migration and invasion and inhibits apoptosis, and is correlated with an unfavorable prognosis of patients with renal cell carcinoma

**DOI:** 10.3892/ijmm.2024.5377

**Published:** 2024-04-24

**Authors:** Liwen Zhao, Jing Quan, Zuwei Li, Xiang Pan, Jingyao Wang, Jinling Xu, Weijie Xu, Xin Guan, Hang Li, Shangqi Yang, Yaoting Gui, Yun Chen, Yongqing Lai

Int J Mol Med 43: 525-534, 2019; DOI: 10.3892/ijmm.2018.3931

Following the publication of the above article, an interested reader drew to the attention of the Editorial Office that, in Fig. 3A on p. 530, two pairs of data panels were overlapping, such that certain of the panels appeared to have been derived from the same original sources where the results from differently performed experiments were intended to have been portrayed.

The authors have examined their original data, and realize that errors associated with data handling/labelling during the preparation of the representative images in Fig. 3A had occurred. The revised version of Fig. 3, showing the correct data for the 'NC/ACHN/Invasion and Migration' data panels, the 'Inhibitor NC/786-O' panel and the 'Inhibitor NC/ACHN/Invasion' panel, is shown on the next page. The authors can confirm that the errors associated with this figure did not have any significant impact on either the results or the conclusions reported in this study, and all the authors agree with the publication of this Corrigendum. The authors are grateful to the Editor of *International Journal of Molecular Medicine* for giving them the opportunity to publish this Corrigendum; furthermore, they apologize to the readership of the Journal for any inconvenience caused.

## Figures and Tables

**Figure 3:**
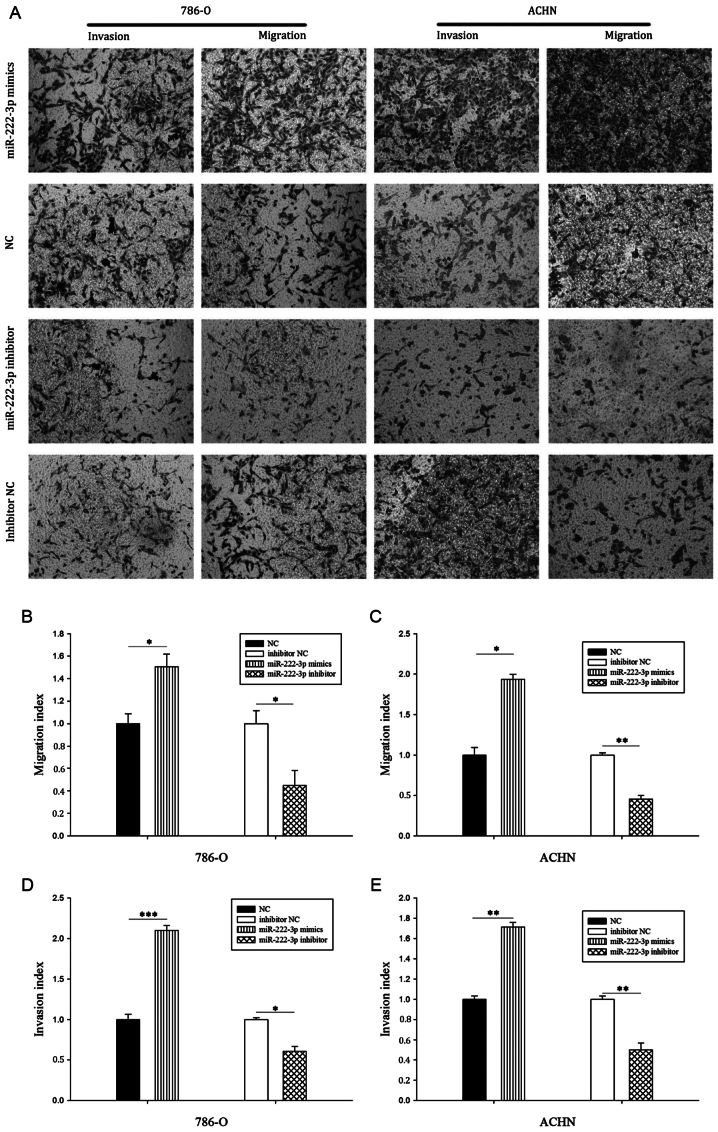
Results of Transwell assays. (A) Representative images of cells in the Transwell migration and invasion assays (magnification, ×100). Overexpression of miR-222-3p enhanced (B) 786-O and (C) ACHN cell migration, whereas knockdown of miR-222-3p inhibited 786-O and ACHN cell migration. Overexpression of miR-222-3p enhanced (D) 786-O and (E) ACHN cell invasion, whereas knockdown of miR-222-3p inhibited 786-O and ACHN cell invasion. ^*^P<0.05, ^**^P<0.01, ^***^P<0.001. miR, microRNA; NC, negative control..

